# Synergistic Effects of Heavy Metals and Pesticides in Living Systems

**DOI:** 10.3389/fchem.2017.00070

**Published:** 2017-10-11

**Authors:** Nitika Singh, Vivek Kumar Gupta, Abhishek Kumar, Bechan Sharma

**Affiliations:** Department of Biochemistry, Faculty of Science, University of Allahabad, Allahabad, India

**Keywords:** contamination, risk assessment, combined interaction, synergistic effect, heavy metal, pesticide

## Abstract

There is a widespread repeated exposure of the population to the pesticides and heavy metals of occupational and environmental origin. Such population is forced to undergo continuous stress imposed by combined exposure of the heavy metals and different classes of the pesticides used in agricultural as well as health practices. The existing reports from several workers have indicated that heavy metals and pesticides in combination may lead more severe impact on the human health when compared to their individual effects. Such a combination of pesticides and heavy metals may also change or influence the detection of exposure. Several studies in past have shown the synergistic toxic effects of heavy metals and pesticides. Such evaluations have revealed the synergistic interactions of various heavy metals and pesticides in animals as well as humans. The aim of the present article is to provide a synthesis of existing knowledge on the synergistic effects of heavy metal and pesticides in living systems. The information included in this article may be useful for different environment protection agencies and policy makers to consider the combined effects of heavy metals and pesticides on humans while designing strategies toward environmental protection and safety regulations about human health.

## Introduction

**Figure 1:**
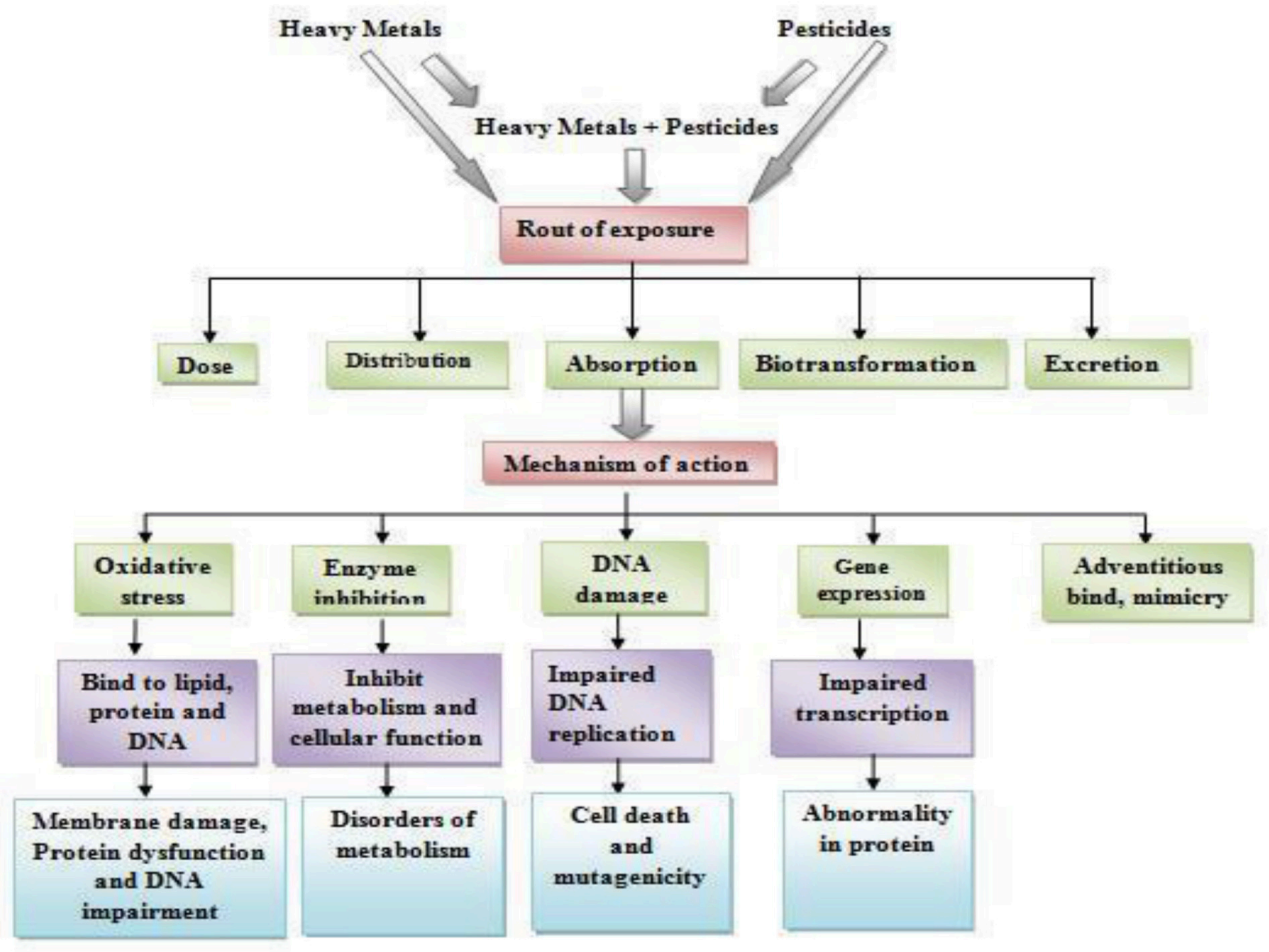
Route of exposure and mechanisms of action of heavy metals and pesticides.

Heavy metals are those inorganic elements which have five times the specific gravity of water (Fergusson, 1990). According to the Agency for Toxic Substances and Disease Registry (2007), arsenic, lead, cadmium (Cd), and mercury have serious health implications among the heavy metals (Csavina et al., 2012; Sharma et al., 2014; Gupta et al., 2015a). Among many heavy metals listed into the d-orbital elements of modern periodic table, arsenic, Cd, mercury, and lead have got prime importance because of their patho-physiological significance as their bioaccumulation in living systems may cause severe damage to the vital organs, namely reproductive systems, nervous system, gastrointestinal tract, and mucous tissues (Sharma et al., 2014; Gupta et al., 2015b). Though the exact mechanism of their pathogenicity is not known but there are reports from various laboratories indicating that the exposure of these heavy metals or their excess accumulation in the body tissues may induce production of free radicals [reactive oxygen species (ROS) and reactive nitrogen species (RNS)] which lead to the production of oxidative stress (OS) (Figure 1; Flora et al., 2008; Sharma et al., 2014; Gupta et al., 2015a; Asmat et al., 2016).

Free radicals have been implicated into DNA damage, oxidation of thiol group(s) of proteins, and lipid peroxidation (LPO) (Figure 1; Valko et al., 2005) which is associated with the onset of various diseases. The Cd and lead are reported to be neurotoxic by inhibiting acetylcholinesterase (AChE) in blood (Gupta et al., 2015b,c) and brain (Gupta et al., 2015b,c), respectively. All heavy metals are toxic in sufficient quantities (Everson et al., 1988; Zukowska and Biziuk, 2008; Taghipour et al., 2014). Because of their presence in our environment and similarity with biochemical activity of some factors involved in the biochemical pathways, lead, mercury, Cd, and arsenic mimics their functions and are of particular interest. Heavy metals produce toxicity by forming complexes with cellular compounds containing sulfur, oxygen, or nitrogen (Aguilera et al., 2017; Kumar et al., 2017; Trost and Tracy, 2017) on entering into our body through food, drinking water, and air. These complexes inactivate or modulate the critical enzyme systems or/protein structures leading to cellular dysfunction and necrosis (Sharma et al., 2014).

On the other hand, the widespread use of pesticides in public health and agricultural programs has caused severe environmental pollution and health hazards, including cases of severe, acute and chronic human poisoning (Satoh, 2006). Pesticides are substances (chemical compounds and naturally occurring phytochemicals) which are used to kill pests in agricultural as well as house hold practices (Damalas and Eleftherohorinos, 2011). They include compounds labeled as insecticides (organochlorines, organophosphates, carbamates, and pyrethroids), rodenticides (arsenic trioxide, barium carbonate and anticoagulants), herbicides (paraquat, diquat, and 2, 4-dichlorophenoxyacetic acid), fungicides (dithiocarbamates, and captan), and fumigants (ethylene dibromide, and methyl bromide (Randall et al., 2013).

According to the Food and Agricultural Organization of the United Sates pesticide are the substances or mixture of substances intended for preventing, destroying or controlling any pest, including vectors of disease, weeds, animals causing harm to the production of crops which may be administered to animals for the control of insects, arachnids or other pests in or on their bodies (Food Agriculture Organization of the United Nations, 2002).

Pesticides have been shown to induce the production of ROS which ultimately leads to the OS (Abdollahi et al., 2004; Sharma et al., 2015). Oxidative stress (OS) occurs when the production of ROS overrides the free radical quenching capacity/antioxidant capacity of the cells, which leads to the damage of cellular biomolecules (nucleic acids, lipids and proteins) involved in structural organization of the cell (Figure 1; Casida and Quistad, 2004; Agrawal and Sharma, 2010). An extensive survey on currently available literatures indicated that pesticide-induced OS has been considered as a possible mechanism of toxicity (Agrawal and Sharma, 2010). The pesticides are known to increase the rate of LPO by altering the activity of both the enzymatic (superoxide dismutase, catalase, and glutathione-S-transferase) and the levels of non-enzymatic (total glutathione, vitamin C and vitamin E) anti-oxidative reserves of the cell and cause OS (Jaiswal et al., 2015, 2016; Figure 1). The impact of pesticide induced OS ranges from tissue injury and aging to the onset of various known/unknown diseases (Agrawal and Sharma, 2010; Agarwal et al., 2012; Chen et al., 2012). The combination exposure of chlorpyrifos (CPF) and Cd has been reported to decrease the mitochondrial potential and induced reactive oxygen species (Xu et al., 2017b).

Some xenobiotics are recalcitrant in nature (Godheja et al., 2016) i.e., they are highly resistant to environmental degradation, such as synthetic organochlorines, natural organic compounds like polyaromatic hydrocarbons. The organochlorines contain carbon, chlorine and hydrogen; the carbon to halide bond being highly resistant to degradation. Therefore, the organochlorines are degraded very slowly and hence remain in the environment or/and inside the organisms after exposure for longer duration (Wandiga, 2001). Carbamates are derived from carbamic acid and used to kill insects/pests (Struger et al., 2016) in a similar fashion as organophosphates. It has been shown that carbofuran is highly neurotoxic and may modulate the functions of acetylcholinesterase (AChE) (Gupta et al., 2016). Organocarbamate pesticides are a class of insecticides which are not broad spectrum in insecticidal function as compared to organophosphates. Most of the carbamates are extremely toxic to hymenoptera (Brunner et al., 2001). It is third-largest order of insects which contains over 150,000 species of arthropods. It comprises the sawflies, wasps, bees, and ants hence the precautions must be taken to avoid exposure to these insects. The pesticide (organocarbamate) exposure to the humans may occur through the inhalation of contaminated air, dermal contact to soils, air and water, drinking water and eating contaminated food (Agrawal and Sharma, 2010). Organophosphates are also a group of wide spectrum pesticides which are reported to be highly neurotoxic and causes several diseases to humans (Agrawal and Sharma, 2010; Gupta and Sharma, 2016). The present article illustrates an updated account of the synergistic effects of heavy metals and the pesticides into different organs of the animals and humans. The manuscript provides a synthesis of existing knowledge on the synergistic effect of heavy metals and pesticides in living system.

## Absorption, distribution and excretion of heavy metals and pesticides exposure

**Figure 2:**
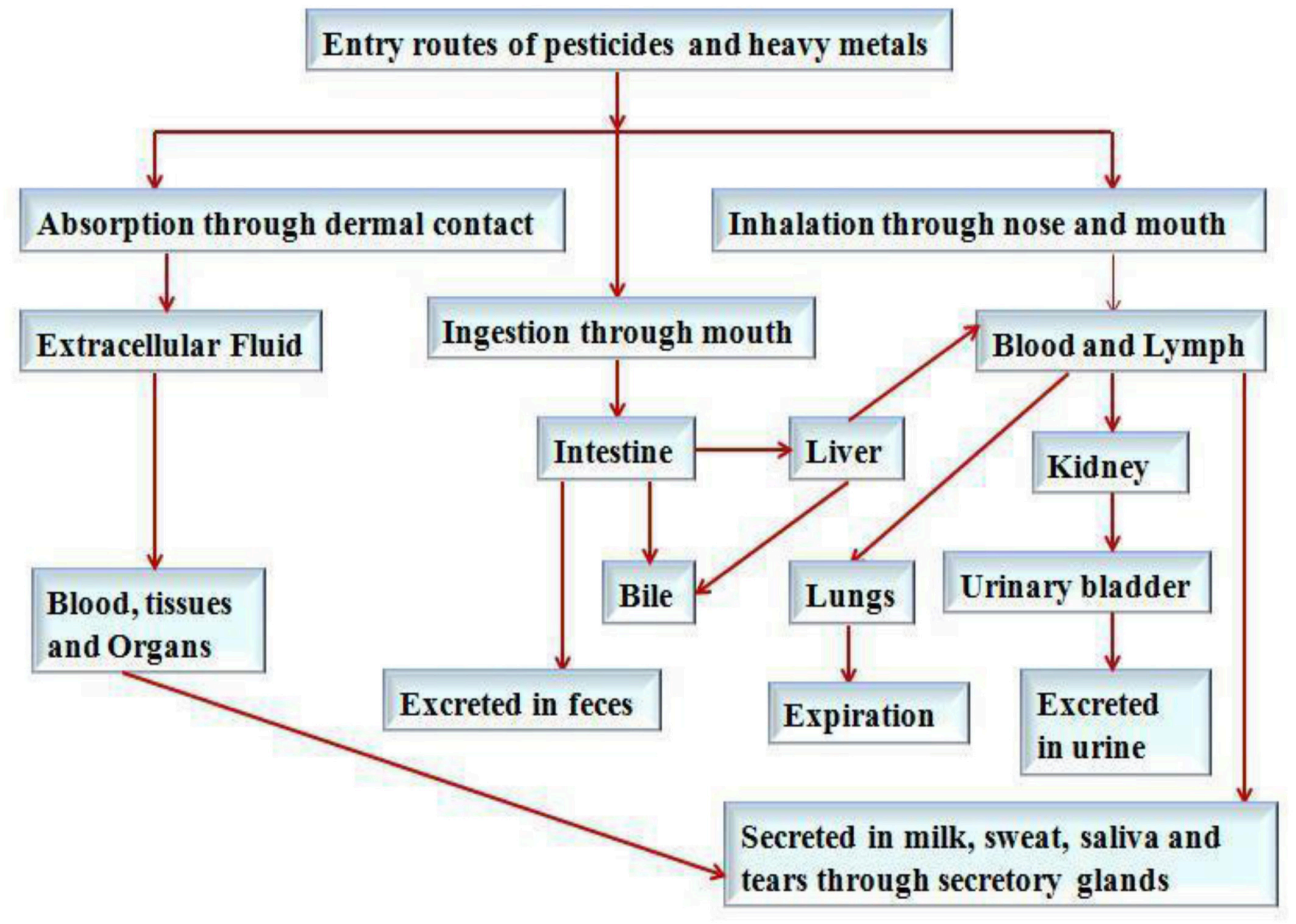
Routes of absorption, distribution and excretion related to the exposure of heavy metals and pesticides in humans.

It has been reported that heavy metals and pesticides mainly enter into the human/animal body through ingestion, such as food materials (Satarug et al., 2003), inhalation and dermal contact, such as emissions of waste material in the form of smoke, dust particle, fume of chemicals from several industrial activities, such as mining, and manufacturing of batteries (Agency for Toxic Substances and Disease Registry, 2007). Other sources of heavy metals a d pesticides exposure to human are agricultural practices, working and smoking in pesticide, heavy metal infested environments, and household practices are the major contributor (International Agency for Research on Cancer, 1993; Paschal et al., 2000). The route of absorption, distribution and excretion related to the exposure of heavy metals and pesticides have been summarized in Figure 2.

## Synergistic effect of heavy metals and pesticides

The living organisms in nature are frequently exposed to a mixture of xenobiotics (heavy metals, pesticides, and toxic gases etc.) simultaneously. The xenobiotic substances have been reported to cause toxicity in animals as well as in key organs of the humans also (Omiecinski et al., 2011; Oesch et al., 2014). Therefore, the combined interactions between xenobiotic substances as well as xenobiotic and animal systems are very important (Oesch et al., 2014). Combined exposure to Cd and ethanol has been shown to produce increased level of norepinephrine in hypothalamus and mid brain of rats in comparison to the rats exposed to only Cd (Flora and Tandon, 1987). The combined effect of heavy metals and pesticides has been listed in Table 1.

**Table 1:** Synergistic effects of heavy metals and pesticides.

**S. No**.	**Synergistic exposure**	**References**	**Effect**
1	Cadmium and Ethanol	Flora and Tandon, 1987	Elevation of nrepinephirine in hypothalamus and mid brain
2	Cadmium and Dimethoate	Institóris et al., 1999, 2001b	Affects relative body weight gain and relative liver weight
3	Lead and Dimethoate	Institóris et al., 1999, 2001b	Affects relative body weight gain, relative liver weight, relative thymus weight and the MCV (mean carpuscular volume) value
4	Cadmium and Propoxur	Institóris et al., 2002	Alter immuno and neurotoxicological function.
5	Cadmium and Diazi on	Creasy, 2001; Adamkovicova et al., 2014	Notable loss of spermatogenic element, disorganization and seminiferous epithelium and lacking maturation of germs cells
6	Mercury and Dimethoate	Institóris et al., 2001b	Alteration in body weight gain, relative liver and kidney weights and in PFC (Igm-plaqueforming cell)
7	Arsenic and Dimethoate	Institóris et al., 2001b	Change in relative liver weight MCV and PFC content of spleen
8	Nickel and Chlorpyrifos	Staal et al., 2007, 2008	Change in molecular fingerprints
9	Arsenic and Lead	Mejia et al., 1997	Alreration in central monoaminogenic system Neurotoxicity and cytotoxicity
10	Mercury and Lead	Mejia et al., 1997	

According to Groten et al. (1997) the exposure of rats to arsenic–lead combinations may produce significant changes in the central monoaminogenic system. Whereas, these alterations were not present when the animals were treated with the same doses of each heavy metals (arsenic and lead) in separate (Mejia et al., 1997). The immunotoxic properties have also been observed due to the combined oral exposures exposure of pesticides and heavy metals in rats (Institóris et al., 1999, 2001a). The interactions occur in both combinations, dimethoate-cadmium (DM-Cd) (Institóris et al., 2002) and dimethoate-lead (DM-Pb) may result into the body weight gain and relative weight gain to liver also (Institóris et al., 1999). The dimethoate-lead (DM-Pb) combination also affected the relative thymus weight and the mean corpuscular volume (MCV) value. These findings showed that the immunotoxic effects of the investigated materials, including their detectability and health consequences can be modified in case of combined exposure (Institóris et al., 1999).

According to the Institóris et al. (2001c), the effects of exposures to cadmium (CdCl2)-propoxur (Pr) as well as rats treated subacutely with dimethoate, As^3+^ and Hg^2+^, has been found to be toxic on the levels of body weight gain, relative organ weights, hematological (RBC, WBC, Ht, MCV, cell content of the femoral bone marrow), immune function, delayed hypersensitivity reaction and neurotoxicity in male Wistar rats. However, a significant interaction between Cd and Pr has also been reported (Institóris et al., 2001c, 2002). The main issue raised by these studies is whether the Lowest Observed Effect Level (LOEL) dose of heavy metals may modulate the toxic effects of Non-Observable Effect Level (NOEL) doses of pesticides (Institóris et al., 2002). Due to having common targets and mechanisms of action, the interaction of Pr and Cd is more likely kinetic. The combined sub-acute exposure of propoxur (carbamate pesticide) and Cd (heavy metal) in rats showed stronger effect than the separate high dose component of the corresponding combinations (Institóris et al., 2002).

The combined exposure of dimethoate (DM) with HgCl2 (Hg), and NaAsO2 (As) has been reported to acquire some abnormality like weight gain of organs (such as weights of brain, thymus, heart, lung, kidneys, adrenals, spleen, testicles), cell count of popliteal lymph node, white blood cell and red blood cells, mean cell volume (MCV) of RBCs, cell content of the femoral bone marrow, IgM-plaque forming cell (PFC) content of the spleen, and delayed hypersensitivity reaction in male rats (Institóris et al., 2001b). The dimethoate-mercury (DM–Hg) combination significantly may cause alterations in the body weight gain, relative liver and kidney weights, and in the plaque forming cell response (Institóris et al., 2001b). However, when dimethoate (DM) as combined with arsenic (As), the significant changes in relative liver weight, value of MCV, and IgM-PFC content of the spleen has been shown (Institóris et al., 2001b).

Chlorpyrifos (CPF) and Nickel (Ni) has been shown to elicit distinct molecular fingerprints and giving rise to a complex transcriptional profile in mixture of both (Dondero et al., 2011; Boatti et al., 2012). Very little studies are known about the dose response relationships between exposures to mixture of xenobiotics on the levels of changes in gene expression. Some studies have reported that the transcriptional patterns found in mixture-exposed samples were largely inherited from the single chemicals (Staal et al., 2007, 2008).

Administration of diazinon and Cd has been reported to cause significant loss of spermatogenic elements (Adamkovicova et al., 2014). A progressive damage in epithelial cells were represented by seminiferous tubules devoid of germ cells and lined by Sertoli cells only (Creasy, 2001) of most seminiferous tubules. The weight of epididymis, testes, and other accessory sex organs, are primary indicators of a possible alteration in androgen status (Biswas et al., 2002; Adamkovicova et al., 2014).

The combined exposure of Cd and diazinon on testis and epididymis has been shown some tubules developing sperm exhibited degeneration and disorganization of seminiferous epithelium, lack in the characteristic maturation of germ cells, and disruption of tight junctions which led to hemorrhaging and edema like conditions in testes (Adamkovicova et al., 2014). Significantly increase in the weight of testis after combined exposure of Cd and diazinon may increase interstitial fluid and damage to the vascular endothelium (Lanning et al., 2002). Furthermore, the dilatation and congestion of the interstitial blood vessels together with necrotizing vasculitis were identified (Fouad et al., 2009). However, the structural perturbations observed in testicular tissue were less expressive than after exposure to diazinon or Cd. The synergistic effect of diazinon and Cd was different from estimates in comparison to the addition of individual xenobiotic (diazinon and Cd) responses (Feron and Groten, 2002).

Similarly, the co-exposure of Cd with nickel (Ni) did not have a synergistic effect on testicular tissues. Combined administration of Cd with nickel may produce fewer pathological alterations than that of Cd alone (İşcan et al., 2002). The synergistic effect of lead (Pb) and mercury (Hg) are extremely neurotoxic and has been reported to be much worse than the single one (Wildemann et al., 2015). The amount of mercury as well as lead sufficient to kill 1% of rats when administered individually, when administered in the combined may kill 100% of rats tested (http://amalgam.org/education/scientific-evidenceresearch/synergistic-effects-of-mercury-other-toxic-exposures/) (Sheets and Sheet, 2018).

In a recent study, the amount of Cd and DM were reported that they are not enough to achieve the toxicological target individually by using cellular pathways. Cd and DM exhibited an additive type of toxicity (Rehman et al., 2017). In animals, IgM antibodies producing PFCs are markedly reduced in number by DM and Cd, as well as by their combinations. Whereas, the oral administration of some pesticides, such as carbaryl, malathion, endosulfan, CPF, quinalphos, and alphamethrin has been reported to suppress the humoral immune response (Wiltrout et al., 1978).

The Cd and CPF induced the protein and LPO, disturbed the total antioxidant capability of cell or organism, and altered ultra-structure of mitochondria in the brain by the oxidative damage. CPF and Cd have been found to lower the potential of mitochondria and generate ROS in SH-SY5Y cells. It has been found that the mixture of CPF and Cd did not display higher toxicity than the sum of the individual treatments. Therefore, it was concluded that they could have a potential antagonistic interaction on the OS induction (Xu et al., 2017b). However, the exact interactions between CPF and Cd are not well-known and needs to be further more investigation regarding their mechanism. Although previous investigation to occurrence and interaction of Cd and CPF simultaneous in environmental medium including food chains show synergistic potential of Toxicity remain elusive thus far. Cd^2+^ and CPF was found to be hepatoxic. By using thin-layer chromatography (TLC) and Nuclear magnetic resonance (NMR) spectroscopy techniques, a novel interaction mechanism between Cd^2+^ and CPF has been reported. In this interaction bonding between Cd^2+^ and nitrogen atom in the pyridine ring of CPF, or the chelation between one Cd^2+^ and two CPF molecules (He et al., 2015). The complex of Cd-CPF showed distinct biological responses and toxicological fates which were different from its parental components. In this study, the joint hepatoxicity of Cd ion and CPF was demonstrated (He et al., 2015). The cause of this hepatoxicity is the formation of Cd-CPF complex which further facilitate the intracellular transport which has been again, reported to be associated with the OS.

The toxicological responses of single substances may be modified by interactions between heavy metals and pesticides which may change the detection limits of their exposure. If the change in detection limits of heavy metals and pesticides occur, it can lead to false-positive and/or false-negative results (Rehman et al., 2017). Further it is necessary to characterize the nature of toxicity on organ system in animals as well as in humans due to the co-exposure of heavy metals and pesticides (Xu et al., 2017a).

In plant system, the synergistic toxicity of heavy metals and pesticides has not been reported well. Very few workers have described the toxicity of heavy metal along with pesticides. (Chen et al., 2004), investigated the combined pollution of 2,4-dichlorophenol (2,4-DCP) along with Cu and Zn. The treatment of 2,4-DCP had limited effect on the dissolution of Cu and Zn in the soil without plant root growth. But the metal species might be changed due to the addition of organic pollutant. Planting with rye grass for 1 month, greatly increased both water soluble Cu and Zn. The increase of water soluble Cu and Zn in the presence of 2,4-DCP was much more than that in the absence of 2,4-DCP, which suggested more attention should be paid to the behavior of heavy metals under combined pollution of organic pollutants, such as pesticides in the planted soil (Chen et al., 2004). The combined effect of more than heavy metal and pesticide in plants have been reported by several workers (Rai et al., 2009; Ong et al., 2013) but in case of combined effect of heavy metal and pesticide in plants sufficient data are not available.

## Mechanism of interactions between heavy metals and pesticides

According to FAO and WHO (2009), the chemical substances to which humans are exposed in the environment have almost infinite number of simple, binary, tertiary and quaternary combinations. The direct experimentation has been found to be unable to resolve the risk assessment issue. According to the recent reports, the research focused on understanding basic science of combination toxicology there are four types of combined effect or interaction being reported which are given below:

1. **Dose addition:** In such type of interactions the toxicity produced through the same mode of action. If there is exposure to a mixture that contains a large number of substances that have the same mechanism of action, may be produced even though the exposures of each substance are too low to elicit a response. According to the dose addition is the basis for recent considerations of pesticides that share the same mode of action.
2. **Response addition:** In this interaction both the substances have different mechanisms of action and individual substance exposure has to be sufficient to cause a response without involvement of other substance. For substance, an active dose of a combination of a neurotoxin and a hepatotoxin produces neurotoxicity and hepatotoxicity and same results were seen when each were given separately. Such interactions are not relevant to the exposures of multiple substances.
3. **Synergism:** Synergism typically occurs when at least one of the components is present to affect the biological system. It occurs when the effect of combination is greater than predicted by the summed activity of each component individually at the same level of exposure that occurs in the mixture. Toxicokinetic interactions occur only when one substance change the metabolism of the other potentially more toxic substance to enhance the internal dose or systemic exposure of the active form of the toxic component (parent compound or metabolite). Such interactions may enhance the activity of the toxic substance which enhances the pesticide activity of the formulation in the target organism.
4. **Antagonism:** In antagonism both the compounds are essential and present at active concentrations. The toxicokinetic/toxicodynamic interactions may result into antagonism. These interactions may decrease the toxicity of the active compound(s). In antagonism two substances occur in which one substance is with low efficacy compete with second substance with high efficacy also known as partial agonist and full agonist, respectively.

For the evaluation of potential of mixture toxicity in reference to the risk assessment of environmental pollutants and its adverse effect on health, a large number of data is required. It is very difficult to decipher the interaction between compounds, because of limited techniques (Chen et al., 2013). There is no any mechanism of interaction has been reported between heavy metals and pesticides. The pesticide and heavy metals may affect the toxicity of each other, which could enhances or decreases the effect of resulting toxic effect and might be responsible for the cumulative effect of both the xenobiotics. The toxicity mechanism of heavy metals and pesticides has been well reported in individual doses separately. However, the synergistic toxicity mechanism of xonobiotics has not been reported clearly. Till now there is no any study found that reported as how heavy metals and pesticides influence or affect the toxicity of each other. Therefore, more study should be focused in this area.

An insecticides and heavy metals contamination show significant health risk to humans under the biological and environmental settings poses (Chen et al., 2013; He et al., 2015). Genotoxic and carcinogenic substances may show their non-linear dose-response relationships. The dose-response assessment should be based on the available dose-response data. The dose metric could be a biomarker for the generation of cancer, and could be validated in relation to the dose. The individual studies of heavy metals and pesticides biotransformation has been studied by many workers. However, there are not enough work has been done in the field of biotransformation of heavy metals and pesticides with respect to the combined study as well as their mechanism by which they affect the toxicity to each other.

## Role of endogenous antioxidant system in reducing toxicity

Endogenous antioxidant system includes both enzymatic and non-enzymatic antioxidants. Some specific antioxidant enzymes, such as Glutathione-S-transferases (GST), glutathione peroxidase (GPx), superoxide dismutase (SOD), catalase, peroxidase and GSH are known to be involved in cellular redox reactions. The endogenous antioxidants (Catalase, GPx, and SOD) play important roles in defense mechanism against OS caused by heavy metals, pesticides as well as their combinations. Lead induced toxicity is mediated by enhancing the production of free radical compounds, such as hydroxyl radical (•OH), superoxide radical (O^−2^), nitric oxide (•NO) and peroxynitrite (ONOO^−^). The major mechanism by which SOD and CAT reduce the OS caused by xenobiotics especially heavy metals, pesticides and their combinations involves decrease in the free radicals concentration by converting the ROS and RNS into nonreactive or less reactive form. The SOD, a metalloenzyme, is responsible for catalyzing the dismutation of the superoxide radical to hydrogen peroxide as a defense mechanism against oxygen toxicity (Simurda et al., 1988). GPx is also involved in this process and eliminate the free radical species by inactivating the hydrogen and lipid peroxides (Flora et al., 2008). The inhibition of phospholipid peroxidation by glutathione peroxidase (GPx), protects membranes from the oxidative damage. It also acts as a H2O2 metabolizing enzyme (Williams et al., 1992; Kwatia et al., 2000). Glutathione-S-transferases (GST) functions to neutralize free radicals causing potential membrane damage via linked catalysis of glutathione (GSH) reduction with detoxification reactions involving thiol-conjugation to xenobiotics (Yan et al., 2008). Catalase, an enzyme responsible for H2O2 metabolism, is functionally replaced by peroxiredoxin (Prx) and GPx in some organism (Sayed et al., 2006).

## Conclusion

The information retrieved from the extensive literature survey indicates that the combinations of pesticides with pesticides, pesticide with heavy metal, and heavy metal to heavy metal acts synergistically and exhibit more toxicity than a single molecule alone. The studies relating heavy metals (Cd and Pb) are also reported to be displaying accelerated toxicological, hematological and immunological indices. In case of humans, the combined exposure of these xenobiotic substances acts in two different ways: firstly, the toxic hazard of a single component could be modified in combined exposures, which can lead to unexpected adverse health consequences. Studies demonstrated that a variety of chemicals may contribute to behavioral disabilities, developmental, and learning impairment. Humans are generally exposed by not only the oral route but also by dermal contact, inhalation, as well as ingestion. Secondly, the delectability of the toxic effects (including immunotoxic) of a single compound can be changed by the interactions with one or more with other heavy metal or other xenobiotics. Thus, the synergistic interactions between pesticides and heavy metals may lead to several health consequences which needed further investigations.

## Author contributions

NS, VKG, and AK wrote the review article, prepared and assembled the Figures and Table; BS critically organized and revised the manuscript by incorporating significant reports.

### Conflict of interest statement

The authors declare that the research was conducted in the absence of any commercial or financial relationships that could be construed as a potential conflict of interest.
